# Enhancing Chinese EFL Students’ Academic Engagement: The Impact of L2 Enjoyment and Academic Motivation

**DOI:** 10.3389/fpsyg.2022.914682

**Published:** 2022-06-10

**Authors:** Xiaotao Wang

**Affiliations:** School of Foreign Languages, Shangqiu Normal University, Shangqiu, China

**Keywords:** L2 enjoyment, academic engagement, Chinese students, EFL classes, academic motivation

## Abstract

Students’ personal, emotional, and psychological traits are perceived to be highly influential in their academic engagement; therefore, many investigations have been conducted into the role of students’ characteristics in their level of engagement. Yet, the role of L2 enjoyment and academic motivation as two instances of students’ emotional traits was disregarded. To narrow this gap, this article aimed to assess the effects of these two constructs on Chinese EFL students’ academic engagement. To accomplish this, three pre-designed scales were virtually administered to 490 Chinese students. Using the Spearman Rho test, significant correlations were discovered among the variables. Further, through regression analysis, the predictive power of dependent variables was also assessed. Chinese students’ academic engagement was proved to be favorably predicted by L2 enjoyment and academic motivation. The implications and limitations are finally discussed.

## Introduction

Students’ academic engagement has been widely viewed as a key to their learning success. That is, many academics strongly believe that students with higher levels of engagement are more likely to succeed in learning environments (Baralt et al., 2016; Kahu and Nelson, 2018). This is why teachers in any instructional-learning environment constantly attempt to improve their students’ classroom engagement. However, the ways in which students’ academic engagement can be improved have remained unknown to a large group of teachers, including EFL teachers (Liu, 2021). Academic engagement, also known as classroom engagement, in a general sense refers to “the quality and quantity of students’ participation or connection with the educational endeavor and hence with the activities, values, individuals, aims, and places that comprise it” (Skinner et al., 2009, p. 497). Academic engagement pertains to students’ active involvement and participation in language courses (Khajavy, 2021). In Mercer’s (2019) words, language learners’ engagement deals with the amount of endeavor and perseverance they willingly dedicate to acquiring a second/foreign language. Likewise, Sang and Hiver (2021) stated that language learners’ engagement has to do with the quantity and quality of attempts they continually devote to language-related activities that directly contribute to L2 success. To underline the significance of academic engagement, Dotterer and Lowe (2011) submitted that engaged learners who eagerly devote more time and energy to performing their educational duties typically outperform their counterparts who do not dedicate sufficient time to accomplishing what they are responsible for. In a similar vein, Yin and Wang (2016) suggested that learners who vigorously participate in classroom tasks and activities are more likely to attain satisfactory outcomes. Likewise, Liem and Chong (2017) posited that energetic and enthusiastic participation in learning contexts enables students to successfully go through the learning process. According to what has been stated concerning the value of academic engagement (Dotterer and Lowe, 2011; Yin and Wang, 2016; Liem and Chong, 2017), identifying the antecedents and determinants of this construct appears to be crucial.

Against this backdrop, many empirical investigations have been conducted into student engagement and its potential antecedents. Among them, several studies have examined the role of teacher-related factors in promoting students’ academic engagement (e.g., Jia et al., 2020; Derakhshan, 2021; Derakhshan et al., 2021; Lu and Mustafa, 2021; Wang and Ye, 2021; Zhou, 2021, among others). Some inquiries have also inspected the function of context-related factors (e.g., Lee et al., 2011; Harbaugh and Cavanagh, 2012; Shernoff et al., 2014; Virtanen et al., 2015, among others). The rest have focused on the effects of students’ personal, psychological, and emotional factors on their academic engagement (e.g., Beri and Stanikzai, 2018; Dincer et al., 2019; Zhang et al., 2020; Chen et al., 2021, among others). Nevertheless, the influence of academic motivation and L2 enjoyment on student academic engagement has rarely been explored. Simply put, limited attention has been dedicated to the function of academic motivation and L2 enjoyment in improving student engagement (Xiong et al., 2015; Oga-Baldwin and Nakata, 2017; Guo, 2021). To narrow this gap, the current study intends to inspect the impact of L2 enjoyment and academic motivation on Chinese students’ engagement in EFL classes.

L2 enjoyment, as a potential antecedent of academic engagement, is a “complex emotion, capturing interacting dimensions of the challenge and perceived ability that reflects the human drive for success in the face of difficult tasks” (Dewaele and MacIntyre, 2016, p. 216). According to Piniel and Albert (2018), L2 enjoyment is a pleasant, stimulating emotion that has been found to favorably influence language learners’ academic outcomes. To them, L2 enjoyment as a stimulating emotion drives language learners to energetically engage in classroom activities. Active participation in the classroom, in turn, contributes to higher academic achievements. Similarly, Dewaele and MacIntyre (2019) argued that L2 enjoyment serves as a powerful incentive for learners, which keeps them motivated and drives them into action. Additionally, relying on control-value theory, Dewaele and Li (2020) stated that L2 enjoyment as an activating feeling promotes language learners’ engagement since learners who enjoy acquiring a second language tend to constantly engage in learning activities.

Academic motivation generally pertains to “individual students’ primary impetus for initiating learning as well as the reason for continuing the prolonged and tedious process of learning” (Ushioda, 2008, p. 20). When it comes to language education, academic motivation refers to language learners’ personal motives to initiate and pursue the laborious process of language learning (Lake, 2013). As put forward by Cheng and Cheng (2012), learners’ increased achievement is tied to their academic motivation. They stated that motivated learners willfully devote adequate time and energy to doing classroom activities, which adds up to desired learning outcomes. In this regard, Feng et al. (2013) also maintained that motivation as a stimulating factor encourages learners to willingly take an active role in learning environments. As noted by Lin et al. (2019), being active in classroom contexts enables learners to achieve the expected outcomes.

Because of the value and importance of L2 enjoyment and academic motivation in learning environments (Cheng and Cheng, 2012; Dewaele and MacIntyre, 2019), a huge number of investigations have been performed on these emotional constructs and their educational outcomes (e.g., Riswanto and Aryani, 2017; Jin and Zhang, 2018; Sivrikaya, 2019; Li, 2020; Tahmouresi and Papi, 2021; Li and Wei, 2022, among others). Yet, the probable consequences of these variables for student academic engagement have remained elusive. Simply put, the extent to which student academic engagement may be affected by L2 enjoyment and academic motivation is an open question. It is due to the fact that the association between students’ academic motivation, L2 enjoyment, and academic engagement has rarely been studied (Xiong et al., 2015; Oga-Baldwin and Nakata, 2017; Guo, 2021). Moreover, to the best of the researcher’s knowledge, no empirical investigation has simultaneously focused on L2 enjoyment and academic motivation to identify their effects on student academic engagement. To address these lacunas, the present investigation aimed to assess the impact of L2 enjoyment and academic motivation on Chinese EFL students’ academic engagement.

## Literature Review

### L2 Enjoyment

The term enjoyment has been generally conceptualized by Li et al. (2018) as “good feelings coming from breaking through homeostatic limits and stretching beyond oneself to accomplish something new or even unexpected, especially in the face of some difficult tasks” (p. 184). Extending this to the context of second language learning, Lee (2020) defined L2 enjoyment as a pleasant feeling experienced by language learners when they acquire or understand something new about the target language. According to Boudreau et al. (2018), the sense of enjoyment that language learners experience in classroom contexts keeps them engaged in the learning process. Further, Li (2020) maintained that those learners who enjoy learning a second or foreign language commonly obtain more desirable outcomes. Building on what different scholars have argued about the educational value of L2 enjoyment (Boudreau et al., 2018; Li, 2020), several researchers have attempted to discover the positive consequences of this construct (e.g., Jin and Zhang, 2018; Lee, 2020; Guo, 2021, among others). Lee (2020), for instance, examined the role of L2 enjoyment in students’ willingness to communicate (WTC). To accomplish this, 647 Korean students were asked to complete two reliable measures of the variables. Assessing Korean students’ answers, the researchers discovered that the L2 enjoyment students experience in their classes can dramatically increase their WTC. In another study, Guo (2021) evaluated the impact of L2 enjoyment on English learners’ engagement. In doing so, “Enjoyment Scale” and “Learner Engagement Scale” were administered to 707 Chinese students. The evaluation of participants’ responses demonstrated that L2 enjoyment can favorably contribute to learners’ engagement.

### Academic Motivation

The term motivation literally means “the need or reason for doing something” (Aiusheeva and Guntur, 2019, p. 453). This definition implies that motivation provides humans with an objective and an orientation to follow (Alizadeh, 2016). More specifically, academic motivation refers to “individual students’ motive to make certain academic decisions, participate in classroom activities, and persist in pursuing the demanding process of learning” (Dörnyei and Ushioda, 2009, p. 4). Student academic motivation is of two types: *“integrative motivation”* and *“instrumental motivation”* (Gardner and MacIntyre, 1991). In the language education domain, students’ integrative motivation pertains to “a need to learn a new language for personal growth and cultural enrichment” (Yu and Downing, 2012, p. 459). On the other hand, students’ instrumental motivation refers to “a desire to acquire a new language for functional or external reasons” (Samad et al., 2012, p. 435). As the findings of previous investigations revealed, student academic motivation is positively associated with academic achievement (Kim and Kim, 2018; Sivrikaya, 2019), willingness to communicate (Joe et al., 2017), and academic engagement (Xiong et al., 2015; Oga-Baldwin and Nakata, 2017). Xiong et al. (2015), for instance, assessed the association of student academic motivation with classroom engagement. To this end, 3,724 students were invited to answer two reliable questionnaires. The data analysis showed a close bond between academic motivation and classroom engagement. Similarly, Oga-Baldwin and Nakata (2017) examined the correlation of student academic motivation with academic engagement. To do this, two pre-designed scales were given to 423 students. Inspecting the correlation of scales, the scholars found a positive relationship between the constructs.

### Academic Engagement

The term academic engagement has been simply characterized by Kuh (2003) as “the time and energy that students put toward their learning” (p. 25). Further, in a more technical manner, Skinner and Pitzer (2012) defined student academic engagement as the extent to which students are enthusiastically committed to and involved in the learning process. Extending this to the language learning context, Finn and Zimmer (2012) described language learners’ academic engagement as how enthusiastically learners are committed to language learning and how vigorously they are immersed in its process. Finally, in light of these definitions, Hiver et al. (2021) conceptualized language learners’ engagement as “the amount (quantity) and type (quality) of learners’ active participation and involvement in language learning tasks/activities” (p. 2). Moving beyond the description of language learner engagement, Zhou et al. (2021) characterized the fundamental dimensions of this construct. They classified the underlying elements of this variable into three basic dimensions of *“behavioral engagement,” “cognitive engagement,”* and *“social engagement.”* Behavioral engagement deals with “individuals’ qualitative behavioral choices in learning.” Cognitive engagement has to do with “learners’ mental activity in the learning process.” Finally, social engagement relates to “relations between interlocutors that support interaction and learning” (Hiver et al., 2021, p. 4). According to SLA theorists (Ariani, 2015; Qureshi et al., 2016), learners’ academic engagement may be remarkably affected by their personal, emotional, and psychological characteristics. Relying on this premise, several researchers have studied the role of learners’ personal, emotional, and psychological traits in their engagement (e.g., Beri and Stanikzai, 2018; Zhang et al., 2020; Chen et al., 2021, among others). Nonetheless, the role of L2 enjoyment and academic motivation as two examples of learners’ emotional traits has remained unclear. This study sought to narrow this lacuna by delving into the role of L2 enjoyment and academic motivation in promoting Chinese EFL students’ academic engagement. To accomplish this, two significant questions were posed:

Are there any significant associations between Chinese EFL students’ academic motivation, L2 enjoyment, and academic engagement?

Do Chinese EFL students’ academic motivation and L2 enjoyment significantly predict their academic engagement?

## Methodology

### Participants

Using convenience sampling approach, 490 Chinese students were chosen from EFL classrooms. For the sake of generalizability, they were selected from five different provinces in China, namely Henan (*N* = 157), Zhejiang (*N* = 124), Guangdong (*N* = 119), Shanghai (*N* = 55), and Jiangsu (*N* = 35). The sample consisted of 460 females and 30 males, ranging in age from 19 to 28 years old (Mean = 23). It comprised 427 BA students (87%), 33 MA students (7%), and 30 Ph.D. candidates (6%). To increase the trustworthiness of the inquiry, they were all informed that their personal information would be kept strictly secret.

### Instruments

#### Classroom Enjoyment Questionnaire

The 6-item version of the “*Classroom Enjoyment Questionnaire* (CEQ),” developed and modified by Lee (2020), was utilized to measure students’ levels of enjoyment in English language classes. The CEQ uses a 5-point Likert scale, ranging in answers from 1 “Strongly disagree” to 5 “Strongly agree.” These are some instances of CEQ’s items: item (2) *“The English teacher is encouraging”* and item (5) *“There is a good atmosphere in English class.”* The reliability of CEQ was found to be 0.89 in this investigation.

#### Student Motivation Scale

To evaluate participants’ levels of motivation, “*Student Motivation Scale*” (Christophel, 1990) was employed. The scale consists of 12 items that participants score on a bipolar scale. Some examples of student motivation scale are: item (3) *“Involved/Uninvolved,”* item (8) *“Uninvigorated/Invigorated,”* and item (12) “Not Fascinated/Fascinated.” The reliability index of student motivation scale for this research was 0.90.

#### Utrecht Work Engagement Scale for Students

To assess the degree to which Chinese EFL students are involved in the process of learning, the “*Utrecht Work Engagement Scale for Students* (UWES-S)” (Schaufeli et al., 2002) was used. The UWES-S comprises 17 items, the answers to which can vary from 1 “Strongly disagree” to 5 “Strongly agree.” The following are two examples of UWES-S’s items: item (9) *“I am enthusiastic about my studies”* and item (14) *“I get carried away when I am studying.”* In the present study, the reliability of UWES-S was calculated to be 0.91.

### Procedure

To begin, the consent form was given to participants through WeChat messenger. Then, using a professional online platform (i.e., Wenjuanxing), the reliable measures of the variables, namely “CEQ,” “Student Motivation Scale,” and “UWES-S” were distributed among them. To ensure the accuracy of responses, all respondents were instructed on how to answer the questionnaires. The answers were fully submitted by the participants in 7 days. To initiate the data analysis, the collected answers were meticulously reviewed to find and eliminate any potential flaws. Afterward, to examine the correlation of academic motivation and L2 enjoyment with student academic engagement, Spearman correlation was employed. Finally, multiple regression analysis was done using SPSS (version 29) to determine how much of the variance in Chinese students’ engagement level can be explained by L2 enjoyment and academic motivation.

## Results

Kolmogorov–Smirnov tests were initially performed to determine whether parametric or non-parametric tests should be used in this study. The results of the tests are shown hereunder (Table 1).

**Table 1:** Test of normality for academic motivation, L2 enjoyment, and academic engagement.

	Kolmogorov–Smirnov			Shapiro–Wilk		
	Statistic	*df*	Sig.	Statistic	*df*	Sig.
L2 enjoyment	0.143	490	0.000	0.915	490	0.000
Academic motivation	0.092	490	0.000	0.936	490	0.000
Academic engagement	0.109	490	0.000	0.921	490	0.000

As delineated in Table 1, the data normality rule was violated for L2 enjoyment (Sig. = 0.000), academic motivation (Sig. = 0.000), and academic engagement (Sig. = 0.000). Accordingly, non-parametric tests were utilized to calculate the possible associations among the variables. To inspect the associations between academic motivation, L2 enjoyment, and academic engagement, Spearman correlation test was conducted. The results of this non-parametric test are represented in the following table (Table 2).

**Table 2:** The associations between academic motivation, L2 enjoyment, and academic engagement.

		L2 enjoyment	Academic motivation	Academic engagement
L2 enjoyment	Correlation coefficient	1.000	0.502^**^	0.585^**^
	Sig. (2-tailed)		0.000	0.000
	*N*	490	490	490
Academic motivation	Correlation coefficient	0.502^**^	1.000	0.418^**^
	Sig. (2-tailed)	0.000		0.000
	*N*	490	490	490
Academic engagement	Correlation coefficient	0.585^**^	0.418^**^	1.000
	Sig. (2-tailed)	0.000	0.000	
	*N*	490	490	490

^**^Correlation is significant at the 0.01 level (2-tailed).

As Table 2 demonstrated, a direct association was found between L2 enjoyment and academic motivation (*r* = 0.502, *n* = 490, *p* = 0.000, *α* = 0.01). The outcomes of Spearman correlation also revealed a direct and strong connection between L2 enjoyment and academic engagement (*r* = 0.585, *n* = 490, *p* = 0.000, *α* = 0.01). Academic motivation was also strongly correlated with academic engagement (*r* = 0.418, *n* = 490, *p* = 0.000, *α* = 0.01). Thus, the higher the level of L2 enjoyment and academic motivation, the greater the amount of academic engagement.

Following that, multiple regression analysis was performed to examine the role of L2 enjoyment and academic motivation in predicting students’ academic engagement. Table 3 delineates the results of regression analysis.

**Table 3:** Model summary for academic motivation, L2 enjoyment, and academic engagement.

Model	*R*	*R* square	Adjusted *R* square	Std. error of the estimate
1	0.675	0.456	0.454	7.71

Predictors: L2 enjoyment, academic motivation.

Dependent variable: academic engagement.

As Table 3 delineated, 45.6% of the variance in Chinese EFL students’ engagement can be explained by L2 enjoyment and academic motivation. That is, both L2 enjoyment and academic motivation were the valuable predictors of Chinese students’ academic engagement.

Table 4 labeled “ANOVA” tested the hypothesis that multiple R in the population equals zero (0). The model reached statistical significance (*F* = (2, 487) = 204.35, Sig. = 0.000, this really means *p* < 0.05).

**Table 4:** ANOVA for academic engagement, L2 enjoyment, and motivation.

Model	Sum of squares	*df*	Mean square	*F*	Sig.	
1	Regression	24352.36	2	12176.18	204.35	0.000
	Residual	29017.67	487	59.585		
	Total	53370.04	489			

Predictors: L2 enjoyment, academic motivation.

Dependent variable: academic engagement.

With regard to the beta column (Table 5), L2 enjoyment was the more significant antecedent of Chinese students’ engagement.

**Table 5:** The coefficients for academic motivation, L2 enjoyment, and academic engagement.

Model	B	Std. error	Beta	*t*	Sig.	
1	(Constant)	18.43	2.25		8.16	0.000
	L2 enjoyment	1.52	0.09	0.62	16.65	0.000
	Academic motivation	0.100	0.03	0.10	2.92	0.000

## Discussion

The initial purpose of this investigation was to measure the degree to which L2 enjoyment and academic motivation are associated with student academic engagement. The Spearman correlation test revealed direct and strong associations, first, between L2 enjoyment and academic engagement, and second, between academic motivation and academic engagement. As to the direct relationship between L2 enjoyment and academic engagement, it is worth noting that this result is in agreement with that of Guo (2021), who discovered a significant and favorable connection between enjoyment and student engagement. Concerning the favorable correlation between academic motivation and academic engagement, it seems encouraging to compare this outcome with that reported by Xiong et al. (2015) who discovered a close relationship between student motivation and classroom engagement. This outcome also accords with Oga-Baldwin and Nakata’s (2017) results, which delineated that learners’ motivation is highly associated with their academic engagement.

As a secondary purpose, this investigation aimed to evaluate the power of L2 enjoyment and academic motivation in predicting Chinese students’ engagement. Put differently, this study sought to determine the extent to which the L2 enjoyment and academic motivation of Chinese students may contribute to their increased engagement. Based on the results of regression analysis, L2 enjoyment was proved to be a powerful predictor of Chinese students’ engagement. It means that Chinese students’ academic engagement highly depends on this pleasant, stimulating emotion. This result lends support to the ideas of Dewaele and MacIntyre (2019), who submitted that L2 enjoyment as an activating feeling drives learners into action. This result is also in line with what Dewaele and Li (2020) have suggested in light of control-value theory. They posited that the pleasant feelings such as joy, satisfaction, and enjoyment learners may experience in learning contexts prompt them to enthusiastically engage in classroom activities. This outcome further corroborates what Zhang and Tsung (2021) have noted regarding the value of L2 enjoyment. They stated that learners who enjoy acquiring a second language are inclined to vigorously participate in classroom activities. Similar to L2 enjoyment, students’ academic motivation was a strong antecedent of their engagement. This result may be readily justified by the fact that motivation as a stimulating factor prompts learners to voluntarily play an active role in learning environments (Feng et al., 2013).

## Conclusion

The present investigation was carried out to evaluate the impact of L2 enjoyment and academic motivation on Chinese EFL students’ engagement. The Spearman correlation test and regression analysis revealed that Chinese students’ enjoyment and academic motivation can greatly influence their academic engagement. Simply put, the higher the level of L2 enjoyment and academic motivation, the greater the amount of academic engagement. Thus, it is logical to infer that those learners who are adequately motivated and those who feel a sense of enjoyment during the learning process are more likely to take part in classroom tasks, exercises, and activities. This appears to be insightful for all English teachers, particularly those who are currently teaching in an EFL setting. With regard to the role of motivation in promoting learners’ engagement, teachers need to provide an ideal learning atmosphere that keeps learners motivated. Given the positive influence of enjoyment on students’ engagement levels, teachers are also expected to satisfy students’ educational needs to provide them with a sense of enjoyment.

Finally, the outcomes of this inquiry are subject to three major limitations. First, the context of this investigation was an EFL country. The results, therefore, need to be generalized to other EFL countries with caution. Since the outcomes of this investigation might not be transferable to ESL contexts, future empirical investigations need to be conducted in other ESL countries. Second, the current investigation employed a quantitative approach to delve into the topic. It would be intriguing to replicate this study using a mixed method approach. Third, in this investigation, the role of age, gender, language proficiency, and educational level was totally neglected, which needs to be considered in future works.

## Data Availability Statement

The original contributions presented in the study are included in the article/supplementary material, further inquiries can be directed to the corresponding author.

## Ethics Statement

The studies involving human participants were reviewed and approved by Shangqiu Normal University Academic and Ethics Committee. The patients/participants provided their written informed consent to participate in this study.

## Author Contributions

The author confirms being the sole contributor of this work and has approved it for publication.

## Conflict of Interest

The author declares that the research was conducted in the absence of any commercial or financial relationships that could be construed as a potential conflict of interest.

## Publisher’s Note

All claims expressed in this article are solely those of the authors and do not necessarily represent those of their affiliated organizations, or those of the publisher, the editors and the reviewers. Any product that may be evaluated in this article, or claim that may be made by its manufacturer, is not guaranteed or endorsed by the publisher.

## References

[ref1] Aiusheeva M. Guntur L. M. F. (2019). Exploring the strategies of raising motivation among ESL students in a non-English speaking context. ELS J. Interdisciplinary Stud. Humanit. 2, 452–464. doi: 10.34050/els-jish.v2i3.7509

[ref2] Alizadeh M. (2016). The impact of motivation on English language learning. Int. J. Res. English Educ. 1, 11–15.

[ref3] Ariani D. (2015). Relationship model of personality, communication, student engagement, and learning satisfaction. Bus. Manag. Educ. 13, 175–202. doi: 10.3846/bme.2015.297

[ref4] Baralt M. Gurzynski-Weiss L. Kim Y. J. (2016). “Engagement with the language: how examining learners’ affective and social engagement explains successful learner-generated attention to form,” in Peer Interaction and Second Language Learning. eds. Sato M. Ballinger S. (Amsterdam: John Benjamin Publishing Company), 209–239.

[ref5] Beri N. Stanikzai M. I. (2018). Self-efficacy beliefs, student engagement and learning in the classroom: a review paper. Am. Int. J. Res. Humanit. Arts Soc. Sci. 22, 213–222.

[ref6] Boudreau C. MacIntyre P. Dewaele J. M. (2018). Enjoyment and anxiety in second language communication: an idiodynamic approach. Stud. Second Lang. Learn. Teach. 8, 149–170. doi: 10.14746/ssllt.2018.8.1.7

[ref7] Chen P. Bao C. Gao Q. (2021). Proactive personality and academic engagement: the mediating effects of teacher-student relationships and academic self-efficacy. Front. Psychol. 12:1824. doi: 10.3389/fpsyg.2021.652994, PMID: 34168588 PMC8217667

[ref8] Cheng C. M. Cheng T. P. (2012). Reflections of the role of motivation on learning English for successful college EFL learners in Taiwan. World J. Educ. 2, 8–14. doi: 10.5430/wje.v2n5p8

[ref9] Christophel D. M. (1990). The relationships among teacher immediacy behaviors, student motivation, and learning. Commun. Educ. 39, 323–340. doi: 10.1080/03634529009378813

[ref10] Derakhshan A. (2021). The predictability of Turkman students’ academic engagement through Persian language teachers’ nonverbal immediacy and credibility. J. Teach. Persian Speaker. Other Lang. 10, 3–26. doi: 10.30479/JTPSOL.2021.14654.1506

[ref11] Derakhshan A. Dolinski D. Zhaleh K. Janebi Enayat M. Fathi J. (2021). A Cross-Cultural Study of Iranian and Polish Higher Education Students’ Academic Engagement in Terms of Teacher Care and Teacher–Student Rapport. Gorgan: Golestan University.

[ref12] Dewaele J. M. Li C. (2020). Emotions in second language acquisition: a critical review and research agenda. A positive psychology perspective on emotions in SLA. Foreign Lang. World 196, 34–49.

[ref13] Dewaele J. M. MacIntyre P. D. (2016). “Foreign language enjoyment and foreign language classroom anxiety. The right and left foot of FL learning?” in Positive Psychology in SLA. eds. MacIntyre P. D. Gregersen T. Mercer S. (Bristol: Multilingual Matters), 215–236.

[ref14] Dewaele J. M. MacIntyre P. D. (2019). “The predictive power of multicultural personality traits, learner and teacher variables on foreign language enjoyment and anxiety,” in Evidence-Based Second Language Pedagogy (London: Routledge), 263–286.

[ref15] Dincer A. Yeşilyurt S. Noels K. A. Vargas Lascano D. I. (2019). Self-determination and classroom engagement of EFL learners: a mixed-methods study of the self-system model of motivational development. SAGE Open 9, 1–15. doi: 10.1177/215824401985391334290901

[ref001] Dörnyei Z. Ushioda E. (eds.) (2009). Motivation, Language Identity and the L2 Self. Bristol: Multilingual Matters., PMID:

[ref16] Dotterer A. M. Lowe K. (2011). Classroom context, school engagement, and academic achievement in early adolescence. J. Youth Adolesc. 40, 1649–1660. doi: 10.1007/s10964-011-9647-5, PMID: 21400208

[ref17] Feng H. Y. Fan J. J. Yang H. Z. (2013). The relationship of learning motivation and achievement in EFL: gender as an intermediated variable. Educ. Res. Int. 2, 50–58.

[ref18] Finn J. D. Zimmer K. S. (2012). “Student engagement: what is it? Why does it matter?” in Handbook of Research on Student Engagement. eds. Christenson S. L. Reschly A. L. Wylie C. (New York, NY: Springer), 97–131.

[ref19] Gardner R. C. MacIntyre P. D. (1991). An instrumental motivation in language study: who says it isn't effective? Stud. Second. Lang. Acquis. 13, 57–72. doi: 10.1017/S0272263100009724

[ref20] Guo Y. (2021). Exploring the dynamic interplay between foreign language enjoyment and learner engagement with regard to EFL achievement and absenteeism: a sequential mixed methods study. Front. Psychol. 12:766058. doi: 10.3389/fpsyg.2021.766058, PMID: 34721246 PMC8554116

[ref21] Harbaugh A. G. Cavanagh R. F. (2012). *Associations between the classroom learning environment and student engagement in learning: A structural equation modelling approach*. Paper Presented at the Joint Australian Association for Research in Education. Sydney, New South Wales.

[ref22] Hiver P. Al-Hoorie A. H. Vitta J. P. Wu J. (2021). Engagement in language learning: A systematic review of 20 years of research methods and definitions. Lang. Teach. Res.:136216882110012. doi: 10.1177/13621688211001289

[ref23] Jia M. Zhang H. Li L. (2020). The power of teacher supportive communication: effects on students’ positive emotions and engagement in learning. Northwest J. Commun. 48, 9–36.

[ref24] Jin Y. Zhang L. J. (2018). The dimensions of foreign language classroom enjoyment and their effect on foreign language achievement. Int. J. Biling. Educ. Biling. 24, 948–962. doi: 10.1080/13670050.2018.1526253

[ref25] Joe H. K. Hiver P. Al-Hoorie A. H. (2017). Classroom social climate, self-determined motivation, willingness to communicate, and achievement: a study of structural relationships in instructed second language settings. Learn. Individ. Differ. 53, 133–144. doi: 10.1016/j.lindif.2016.11.005

[ref26] Kahu E. R. Nelson K. (2018). Student engagement in the educational interface: understanding the mechanisms of student success. High. Educ. Res. Dev. 37, 58–71. doi: 10.1080/07294360.2017.1344197

[ref27] Khajavy G. H. (2021). “Modeling the relations between foreign language engagement, emotions, grit and reading achievement,” in Student Engagement in the Language Classroom. eds. Hiver P. Al-Hoorie A. H. Mercer S. (Bristol: Multilingual Matters), 241–259.

[ref28] Kim Y. K. Kim T. Y. (2018). An investigation on male high school students’ motivation and achievement in English learning. English Teach. 73, 135–160. doi: 10.15858/engtea.73.1.201803.135

[ref29] Kuh G. D. (2003). What we’re learning about student engagement from NSSE: benchmarks for effective educational practices. Change Magazine Higher Learn. 35, 24–32. doi: 10.1080/00091380309604090

[ref30] Lake J. (2013). “Positive L2 self: linking positive psychology with L2 motivation,” in Language Learning Motivation in Japan. eds. Apple M. T. Silva D. D. Fellner T. (Bristol: Multilingual Matters), 225–244.

[ref31] Lee J. S. (2020). The role of grit and classroom enjoyment in EFL learners’ willingness to communicate. J. Multiling. Multicult. Dev. 1, 1–17. doi: 10.1080/01434632.2020.1746319

[ref32] Lee J. C. K. Zhang Z. Song H. (2011). Effects of quality of school life and classroom environment on student engagement: a Chinese study. Educ. Pract. Theory 33, 5–27. doi: 10.7459/ept/33.1.02

[ref33] Li C. (2020). A positive psychology perspective on Chinese EFL students’ trait emotional intelligence, foreign language enjoyment and EFL learning achievement. J. Multiling. Multicult. Dev. 41, 246–263. doi: 10.1080/01434632.2019.1614187

[ref34] Li C. Jiang G. Dewaele J. M. (2018). Understanding Chinese high school students’ foreign language enjoyment: validation of the Chinese version of the foreign language enjoyment scale. System 76, 183–196. doi: 10.1016/j.system.2018.06.004

[ref35] Li C. Wei L. (2022). Anxiety, enjoyment, and boredom in language learning amongst junior secondary students in rural China: how do they contribute to L2 achievement? Stud. Second. Lang. Acquis., 1–16. doi: 10.1017/S0272263122000031

[ref36] Liem G. A. D. Chong W. H. (2017). Fostering student engagement in schools: international best practices. Sch. Psychol. Int. 38, 121–130. doi: 10.1177/0143034317702947

[ref37] Lin L. C. Hung I. Chen N. S. (2019). The impact of student engagement on learning outcomes in a cyber-flipped course. Educ. Technol. Res. Dev. 67, 1573–1591. doi: 10.1007/s11423-019-09698-9

[ref38] Liu S. (2021). Toward the role of L2 enjoyment in EFL students’ academic motivation and engagement. Front. Psychol. 12:822588. doi: 10.3389/fpsyg.2021.822588, PMID: 35153938 PMC8825799

[ref39] Lu Q. Mustafa Z. (2021). Toward the impact of EFL teachers' self-efficacy and collective efficacy on students’ engagement. Front. Psychol. 12:744586. doi: 10.3389/fpsyg.2021.744586, PMID: 34589035 PMC8473623

[ref40] Mercer S. (2019). “Language learner engagement: setting the scene,” in Second Handbook of English Language Teaching. ed. Gao X. (New York, NY: Springer), 1–19.

[ref41] Oga-Baldwin W. Q. Nakata Y. (2017). Engagement, gender, and motivation: a predictive model for Japanese young language learners. System 65, 151–163. doi: 10.1016/j.system.2017.01.011

[ref002] Piniel K. Albert A. (2018). Advanced learners’ foreign language-related emotions across the four skills. Stud. Second Lang. Learn. Teach. 8, 127–147.

[ref42] Qureshi A. Wall H. Humphries J. Balani A. B. (2016). Can personality traits modulate student engagement with learning and their attitude to employability? Learn. Individ. Differ. 51, 349–358. doi: 10.1016/j.lindif.2016.08.026

[ref43] Riswanto A. Aryani S. (2017). Learning motivation and student achievement: description analysis and relationships both. Int. J. Couns. Educ. 2, 42–47. doi: 10.23916/002017026010

[ref44] Samad A. A. Etemadzadeh A. Far H. R. (2012). Motivation and language proficiency: instrumental and integrative aspects. Procedia Soc. Behav. Sci. 66, 432–440. doi: 10.1016/j.sbspro.2012.11.287

[ref45] Sang Y. Hiver P. (2021). “Engagement and companion constructs in language learning: conceptualizing learners’ involvement in the L2 classroom,” in Student Engagement in the Language Classroom. eds. Hiver P. Al-Hoorie A. H. Mercer S. (Clevedon: Multilingual Matters), 17–37.

[ref46] Schaufeli W. B. Salanova M. González-Romá V. Bakker A. B. (2002). The measurement of engagement and burnout: a two sample confirmatory factor analytic approach. J. Happiness Stud. 3, 71–92. doi: 10.1023/A:1015630930326

[ref47] Shernoff D. J. Tonks S. M. Anderson B. (2014). The impact of the learning environment on student engagement in high school classrooms. Teach. Coll. Rec. 116, 166–177. doi: 10.1177/016146811411601315

[ref48] Sivrikaya A. H. (2019). The relationship between academic motivation and academic achievement of the students. Asian J. Educ. Train. 5, 309–315. doi: 10.20448/journal.522.2019.52.309.315

[ref49] Skinner E. A. Kindermann T. A. Furrer C. J. (2009). A motivational perspective on engagement and disaffection: conceptualization and assessment of children's behavioral and emotional participation in academic activities in the classroom. Educ. Psychol. Meas. 69, 493–525. doi: 10.1177/0013164408323233

[ref50] Skinner E. A. Pitzer J. R. (2012). “Developmental dynamics of student engagement, coping, and everyday resilience,” in Handbook of Research on Student Engagement. eds. Christenson S. L. Reschly A. L. Wylie C. (New York, NY: Springer), 21–44.

[ref51] Tahmouresi S. Papi M. (2021). Future selves, enjoyment and anxiety as predictors of L2 writing achievement. J. Second. Lang. Writ. 53:100837. doi: 10.1016/j.jslw.2021.100837

[ref52] Ushioda E. (2008). “Motivation and good language learners” in Lessons From Good Language Learners. ed. Griffiths C. (Cambridge: Cambridge University Press), 19–34.

[ref53] Virtanen T. E. Lerkkanen M. K. Poikkeus A. M. Kuorelahti M. (2015). The relationship between classroom quality and students’ engagement in secondary school. Educ. Psychol. 35, 963–983. doi: 10.1080/01443410.2013.822961

[ref54] Wang F. Ye Z. (2021). On the role of EFL/ESL teachers’ emotion regulation in students' academic engagement. Front. Psychol. 12:758860. doi: 10.3389/fpsyg.2021.758860, PMID: 34690902 PMC8527877

[ref55] Xiong Y. Li H. Kornhaber M. L. Suen H. K. Pursel B. Goins D. D. (2015). Examining the relations among student motivation, engagement, and retention in a MOOC: a structural equation modeling approach. Global Educ. Rev. 2, 23–33.

[ref56] Yin H. Wang W. (2016). Undergraduate students’ motivation and engagement in China: an exploratory study. Assess. Eval. High. Educ. 41, 601–621. doi: 10.1080/02602938.2015.1037240

[ref57] Yu B. Downing K. (2012). Determinants of international students’ adaptation: examining effects of integrative motivation, instrumental motivation and second language proficiency. Educ. Stud. 38, 457–471. doi: 10.1080/03055698.2011.643111

[ref58] Zhang X. Chen G. Xu B. (2020). The influence of group big-five personality composition on student engagement in online discussion. Int. J. Inf. Educ. Technol. 10, 744–750. doi: 10.18178/ijiet.2020.10.10.1452

[ref59] Zhang L. Tsung L. (2021). Learning Chinese as a second language in China: positive emotions and enjoyment. System 96:102410. doi: 10.1016/j.system.2020.102410

[ref60] Zhou X. (2021). Toward the positive consequences of teacher-student rapport for students' academic engagement in the practical instruction classrooms. Front. Psychol. 12:759785. doi: 10.3389/fpsyg.2021.759785, PMID: 34675860 PMC8525607

[ref61] Zhou S. Hiver P. Al-Hoorie A. H. (2021). “Measuring L2 engagement: a review of issues and applications,” in Student Engagement in the Language Classroom. eds. Hiver P. Al-Hoorie A. H. Mercer S. (Clevedon: Multilingual Matters), 75–98.

